# Predictors of Postprandial Hypoglycemia After Gastric Bypass Surgery: a Retrospective Case-Control Study

**DOI:** 10.1007/s11695-021-05277-1

**Published:** 2021-02-23

**Authors:** Elric Zweck, Matthias Hepprich, Marc Y. Donath

**Affiliations:** 1 Clinic of Endocrinology, Metabolism and Diabetes, University Hospital Basel, Petersgraben 4, 4031 Basel, Switzerland; 2 Institute for Clinical Diabetology, German Diabetes Center, Leibniz Center for Diabetes Research at Heinrich Heine University, Auf´m Hennekamp 65, 40225 Düsseldorf, Germany; 3 Division of Cardiology, Pulmonology and Vascular Medicine, Medical Faculty, Heinrich Heine University Düsseldorf, Moorenstrasse 5, 40225 Düsseldorf, Germany; 4 Clinic of Endocrinology and Metabolic Disorders, Cantonal Hospital Olten, Basler Strasse 150, 4600 Olten, Switzerland

**Keywords:** Postprandial hypoglycemia, Late-dumping, Bariatric surgery, Mixed-meal test, Complications

## Abstract

**Background:**

Postprandial hypoglycemia after bariatric surgery is an exigent disorder, often impacting the quality of life. Distinguishing clinically relevant hypoglycemic episodes from symptoms of other origin can be challenging. Diagnosis is demanding and often requires an extensive testing such as prolonged glucose tolerance or mixed-meal test. Therefore, we investigated whether baseline parameters of patients after gastric bypass with suspected hypoglycemia can predict the diagnosis.

**Methods:**

We analyzed data from 35 patients after gastric bypass with suspected postprandial hypoglycemia and performed a standardized mixed-meal test. Hypoglycemia was defined by the appearance of typical symptoms, low plasma glucose, and relief of symptoms following glucose administration. Parameters that differed in patients with and without hypoglycemia during MMT were identified and evaluated for predictive precision using receiver operating characteristic (ROC) areas under the curve (AUC).

**Results:**

Out of 35 patients, 19 (54%) developed symptomatic hypoglycemia as a result of exaggerated insulin and C-peptide release in response to the mixed-meal. Hypoglycemic patients exhibited lower glycosylated hemoglobin A1c (HbA_1c_) and higher absolute and relative weight loss from pre-surgery to study date. HbA1c and absolute weight loss alone could achieve acceptable AUCs in ROC analyses (0.76 and 0.72, respectively) but a combined score of absolute weight loss divided by HbA1c (0.78) achieved the best AUC.

**Conclusions:**

HbA1c and weight loss differed in patients with and without symptomatic hypoglycemia during mixed-meal test. These baseline parameters could be used for screening of postprandial hypoglycemia in patients after gastric bypass and may facilitate the selection of patients requiring further evaluation.

**Graphical abstract:**

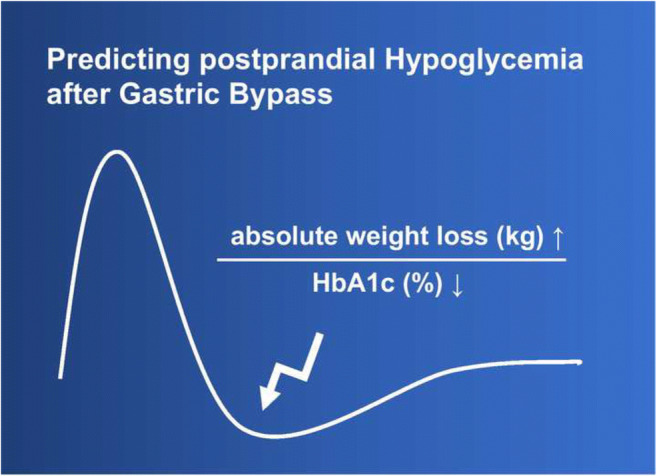

**Supplementary Information:**

The online version contains supplementary material available at 10.1007/s11695-021-05277-1.

## Introduction

Bariatric surgery is an effective treatment modality for obesity [1–3] with confirmed long-term safety and overall benefit regarding weight loss, components of the metabolic syndrome, quality of life, and survival [4–6].

Nevertheless, up to a third of postbariatric patients report symptoms of postprandial hyperinsulinemic hypoglycemia [7], whereas some studies report even higher prevalence rates in standardized test settings [8, 9] and severe hypoglycemic episodes occurring in less than 12% of patients [10, 11]. However, the exact prevalence remains unknown. Affected patients may experience neuroglycopenic and vegetative symptoms with different intensity typically within 3 h after carbohydrate intake [12]. Postbariatric hypoglycemia may lead to an impairment of quality of life and to increased food intake with subsequent weight regain [13].

Known risk factors for hypoglycemia in postbariatric patients are younger age, female gender, greater postoperative loss of weight, and pre-operative high insulin sensitivity [14–16]. The diagnosis of hypoglycemia is often demanding and requires fulfillment of Whipple’s triad often established with provocation testing [13, 14, 16–18]. These cumbersome and cost-intense tests require constant observation of the patient for several hours by health care professionals. Therefore, they should ideally only be performed in patients with a high a priori chance of hypoglycemia [17]. Furthermore, the lack of a reliable and simple screening tool may partly explain underdiagnosis of hypoglycemia in postbariatric patients [12].

We, therefore, investigated whether baseline parameters of postbariatric patients can predict the occurrence of symptomatic hypoglycemia during a mixed-meal test.

## Methods

### Data Source and Study Population

We evaluated all data of patients after bariatric surgery who underwent mixed-meal testing because of symptoms suspicious for postprandial hypoglycemia at the Clinic for Endocrinology, Diabetes and Metabolism of the University Hospital Basel between May 2017 and October 2019, where approximately 100 patients undergo bariatric surgery annually. Patients are regularly followed up after bariatric surgery in our center. Participating patients presented with history of hypoglycemic symptoms and were therefore screened for this condition at our institution. In some of these patients, the provocation tests were also used as screening for a clinical trial (clinicaltrials.gov NCT03200782). Patients that underwent a MMT but also had diabetes mellitus (*n* = 1), gastric sleeve surgery (*n* = 1), or nesidioblastosis (*n* = 1) were excluded from analyses. All patients gave written informed consent for the use of their data.

### Standardized Liquid Meal Test

Following an overnight fast, patients ingested a liquid 300 ml of mixed-meal drink containing 450 kcal and 60 g of carbohydrates (Ensure® Plus). Patients had to rest in a 45° upright position for the whole test. Heart rate and blood pressure were assessed at baseline. Every 30 min bedside glucose was measured, and venous blood samples were drawn to assess glucose, insulin, and C-peptide. Occurrence of hypoglycemic symptoms was monitored by checking for typical symptoms according to the Edinburgh Hypoglycemia scale and neurocognitive questions comprising repetitive questions for date of birth, current date, serial subtraction of seven-test, repeating words, and backward spelling of words. In patients presenting with symptomatic hypoglycemia during the test, 10 g of glucose were administered intravenously or orally depending on severity of the symptoms by a physician. If symptoms persisted, glucose was given repetitively until symptoms resolved and blood glucose normalized. The test was terminated after two consecutive measurements of blood glucose rising again after the initial postprandial drop or after 210 min without any hypoglycemic symptoms.

At baseline, insulin resistance was estimated using Homeostasis Model Assessment (HOMA and HOMA2) [19]. Several additional indices for insulin sensitivity and insulin secretion using the data assessed during the MMT were estimated: Whole-body Insulin Sensitivity Index, Oral Glucose Insulin Sensitivity, Predicted M-value, Insulin Secretion Index I, and Insulin Secretion Index II [20–24].

### Statistical Analyses

Primary outcome of interest was the occurrence of symptomatic hypoglycemia during a standardized MMT, defined as low plasma glucose (< 3.4 mM) concurrent with typical symptoms, which can be relieved by glucose administration (Whipple’s triad). Statistical analyses were conducted with SPSS® Statistics 25.0.0.2 (IBM®, Armonk, NY, USA) and Python 3.7.4 (Python Software Foundation, DE, USA) with publicly available software packages (pandas, NumPy, Matplotlib, scikit-learn, tableOne, statsmodels, SciPy). In figures, data are presented as means ± standard error of the mean and those given in tables are presented as median with interquartile range. Groups were compared using Kruskal-Wallis test. Receiver operating characteristic (ROC) curves were calculated for all continuous variables in the dataset. Areas under the curves (AUCs) of these ROC curves were calculated to identify most predictive variables. Univariate ROC analysis was chosen over multivariate logistic regression due to (i) the sample size, which was adequately powered for an univariate ROC with an alpha of 0.05 and a beta of 0.2, but not sufficient for a multivariate linear model, and (ii) due to the easy clinical applicability of ROC due to its intrinsic advantage of providing cutoffs for the examined predictors. Univariate logistic regression was additionally performed to provide odd ratios for all continuous variables. For the creation of the score, highly co-linear variables with a Spearman rank correlation coefficient > 0.6 were excluded by removing the variable with the smaller AUC in the ROC curve. Statistical significance threshold was *p* < 0.05.

## Results

### Baseline Characteristics of Patients

Thirty-five patients were included in the analyses, 19 patients (54%) presented with hypoglycemia, whereas in 16 patients (46%), the suspicion of hypoglycemia could not be confirmed by the MMT. Baseline characteristics of both groups are depicted in Table 1. Both groups exhibited comparable age, sex distribution, and BMI. All patients had undergone Roux-en-Y gastric bypass surgery. At baseline, the only significant differences between the groups were HbA_1c_ and absolute and relative weight loss as compared to the weight pre-surgery (Table 1).

**Table 1:** Participants’ characteristics for patients without symptomatic hypoglycemia and with hypoglycemia after gastric bypass surgery

Variable	Unit	Non-hypoglycemia	Hypoglycemia	*p*	Missing
*n*		16	19		
Age	years	40.8 (37.5, 47.8)	42.9 (35.4, 51.0)	0.947	0
Years since surgery	years	4.5 (2.9, 6.4)	3.9 (1.7, 5.5)	0.175	0
Sex (female)		14 (87.5)	16 (84.2)	1.000	0
T2DM pre-surgery		2 (14.3)	2 (12.5)	1.000	5
T2DM current		0 (0)	0 (0)	1	0
Weight pre-surgery	kg	109.5 (103.8, 121.5)	116.2 (107.5, 129.5)	0.296	0
Weight current	kg	78.5 (69.0, 91.0)	78.7 (68.8, 82.5)	0.619	0
Absolute weight loss	kg	29.0 (25.0, 35.2)	39.0 (31.6, 53.5)	0.024	0
Relative weight loss	%	28.3 (23.1, 30.8)	35.0 (27.7, 45.6)	0.024	0
BMI pre-surgery	kg/m^2^	39.4 (38.1, 42.6)	43.4 (39.8, 45.6)	0.132	0
BMI current	kg/m^2^	28.3 (25.8, 31.8)	28.2 (24.7, 30.3)	0.436	0
Change in BMI	kg/m^2^	10.9 (9.7, 12.8)	14.5 (10.9, 19.5)	0.028	0
Relative change in BMI	%	28.3 (23.1, 30.8)	35.0 (27.7, 45.6)	0.024	0
Systolic blood pressure	mmHg	119.0 (115.5, 131.5)	109.0 (100.0, 117.0)	0.060	10
Diastolic blood pressure	mmHg	80.0 (68.8, 82.5)	71.0 (68.0, 81.0)	0.462	10
Heart rate	min^−1^	74.5 (66.0, 81.2)	72.0 (68.0, 80.0)	0.723	10
Baseline glucose	mmol/l	4.7 (4.4, 4.8)	4.5 (4.5, 4.7)	0.485	12
Baseline insulin	mU/l	5.5 (3.5, 8.5)	6.0 (4.3, 9.6)	0.832	16
Baseline C-peptide	pmol/l	609.0 (519.8, 739.5)	693.5 (579.8, 740.0)	0.512	17
HbA_1c_	%	5.3 (5.0, 5.7)	4.9 (4.7, 5.2)	0.009	0
Hemoglobin	g/l	133.0 (126.8, 145.5)	126.5 (122.2, 135.8)	0.097	1
C-reactive protein	mg/l	0.6 (0.4, 2.0)	0.7 (0.3, 1.6)	0.637	1
Glomerular filtration rate	ml/min/1.7	99.0 (86.2, 112.0)	103.5 (99.2, 111.2)	0.333	1
HOMA-IR		1.2 (0.7, 1.8)	1.3 (0.9, 2.1)	0.401	4
HOMA-beta		81.3 (53.0, 124.2)	127.7 (84.0, 159.4)	0.139	4
HOMA2-IR		1.3 (1.2, 1.5)	1.4 (1.1, 1.5)	0.808	4
HOMA2-beta		132.1 (110.6, 150.1)	138.8 (123.2, 153.2)	0.612	4

p values were calculated with Kruskal-Wallis test or Fisher’s test. HbA_1c_, glycosylated hemoglobin A_1c_; HOMA, Homeostasis Model Assessment; IR, insulin resistance; PBH, postbariatric hypoglycemia; T2DM, type 2 diabetes mellitus

### Glucose Metabolism During Mixed-Meal Test

**Fig. 1:**
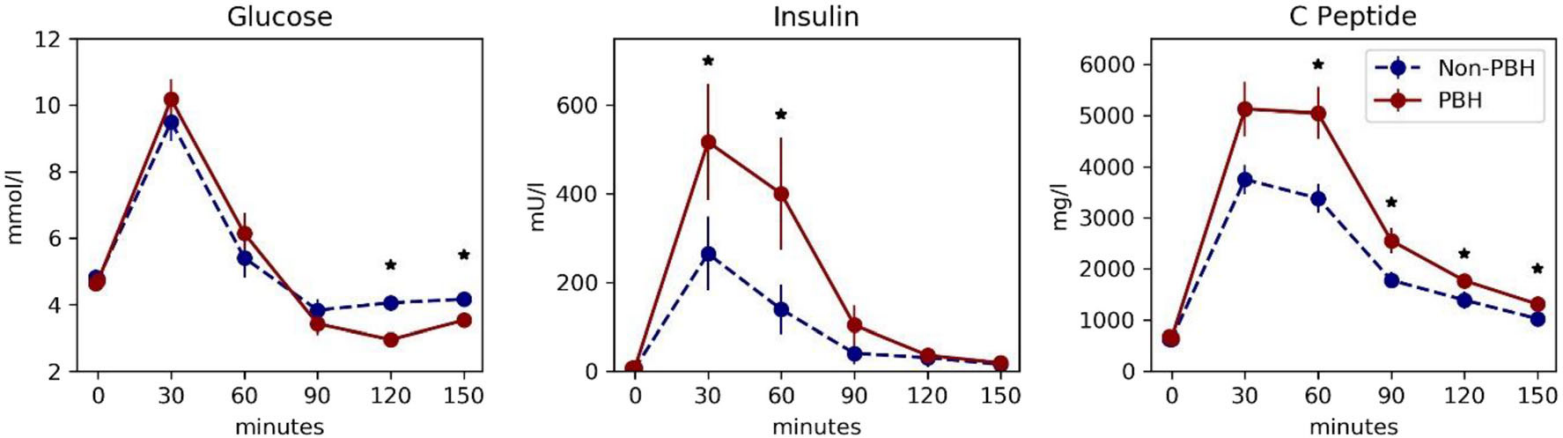
Profiles for glucose, insulin, and C-peptide during a standardized mixed-meal test. Data are presented as mean ± standard error of the mean. *n* = 29–36 for each time point. *p < 0.05 with Kruskal-Wallis test. Red solid line patients with symptomatic hypoglycemia and dashed blue line patients without symptomatic hypoglycemia after gastric bypass surgery

We compared plasma glucose, insulin, and C-peptide levels during the MMT (Fig. 1). Meal intake induced an exaggerated immediate stimulation in insulin and C-peptide leading to hypoglycemic levels after 120 min in patients with hypoglycemia compared to patients without hypoglycemia. Insulin resistance did not differ between groups, whereas insulin secretion assessed by HOMA-beta and insulin secretion indices was numerically increased, without reaching statistical difference (Table 2).

**Table 2:** Indices of insulin secretion and insulin sensitivity during mixed-meal tests

Variable	Non-hypoglycemia	Hypoglycemia	*p*
*n*	12	18	
Whole-body Insulin Sensitivity Index [22]	103.5 (91.1, 120.6)	72.8 (43.1, 115.6)	0.099
Oral Glucose Insulin Sensitivity [23]	475.5 (457.4, 510.1)	483.0 (393.1, 531.9)	0.966
Predicted M-value [24]	2.4 (2.1, 2.4)	2.6 (2.2, 2.7)	0.225
Insulin Secretion Index I [20]	41.5 (35.7, 66.7)	55.2 (40.8, 95.6)	0.310
Insulin Secretion Index II [21]	20.5 (15.5, 31.4)	32.3 (20.1, 48.8)	0.189

*p* values were calculated with Kruskal-Wallis test

### Prediction of Occurrence of Postprandial Hypoglycemia

**Fig. 2:**
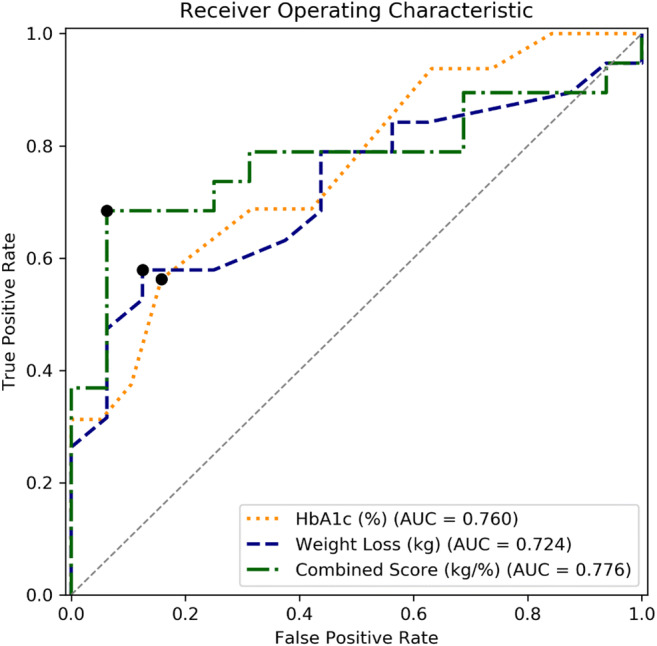
Receiver operating characteristic curves. Curves are plotted with local optima, defined as the maximum of the true positive rate minus the false positive rate. The HbA_1c_ curve is displayed inversely for comparison. The combined score was calculated as the ratio of absolute postoperative weight loss (kg) by HbA_1c_ (%)

Based on our findings from univariate group comparisons and all continuous variables’ AUCs from univariate ROC curves (Supplemental Table 1), we identified HbA_1c_ and absolute weight loss as the most predictive parameters for occurrence of postprandial hypoglycemia in our dataset. Both variables achieved acceptable performance in ROC analyses (Fig. 2) with optimal cutoffs for HbA_1c_ at 5.3% (sensitivity 56%, specificity 84%) and weight loss of 38.2 kg (sensitivity 58%, specificity 88%), respectively (Supplemental Table 2). However, a combined score of the two variables, calculated as the ratio of absolute weight loss (in kg) to current HbA_1c_ (in %), achieved the highest AUC of 0.784, using 7.2 (kg/%) as a cutoff and achieving a sensitivity of 68% and a specificity of 94% at the local maximum.

## Discussion

It is presumed that the overall prevalence of hypoglycemia in postbariatric patients is underestimated [7, 9]. One reason for this is the lack of standardized, practicable, and affordable screening tests. We demonstrate that in patients with suspected hypoglycemia, a score using HbA1c and the postoperative weight loss is able to identify patients at risk for symptomatic hypoglycemia during an MMT. We further depict baseline and in-test differences of patients with and without hypoglycemia. These observations might ease future screening for hypoglycemia in patients after gastric bypass surgery.

By performing an MMT with repeated measurement of glucose, insulin, and C-peptide, we could confirm the pathognomonic pattern of postbariatric hypoglycemia in our study cohort: in patients with hypoglycemia, insulin and C-peptide increased drastically compared to patients without hypoglycemia, whereas glucose levels lowered markedly in the hypoglycemia group. With both groups starting from similar baseline values, these data also confirm the exclusively postprandial occurrence of hyperinsulinemia in the hypoglycemia group. These findings contrast previous studies reporting insignificant differences in postprandial glucose and insulin profiles between postbariatric patients with and those without postprandial symptoms [25, 26]. One possible explanation might be the larger number of patients in our cohort.

We used different indices for an estimation of insulin secretion capacity and insulin sensitivity, which did not indicate significant differences between patients with and without hypoglycemia regarding these parameters. This contrasts findings from a recent study by Raverdy et al. that suggested beta-cell function and insulin sensitivity might differentiate patients with and without hypoglycemia [14]. This difference to our study might result from the use of an oral glucose tolerance test in their study in contrast to a mixed-meal test in ours. Interpreting this is difficult due to the fact that none of these indices have been designed for postbariatric patients, hinting towards the need for more precise tools in these patients.

The outcome of interest for this study was the occurrence of late dumping, i.e., Whipple’s triad, during a standardized MMT. This way, our study population’s hypoglycemia status is well-characterized as opposed to large cohort or register studies, which define hypoglycemia by past diagnosis. In contrast to previous studies, age, sex, and fasting glucose did not distinguish patients with and without hypoglycemia in our study [11, 16, 27]. Higher preoperative insulin sensitivity may be another risk factor but was not assessed in this cohort [11]. In our trial cohort, lower HbA_1c_ and higher absolute weight loss clearly distinguished patients with and without PBH. Low HbA_1c_ could be explained by multiple episodes of hypoglycemia during the months before testing; however, it has not been reported in hypoglycemia patients yet. Greater weight loss in hypoglycemia patients has been reported in previous studies [14, 15, 27, 28] and might overstrain metabolic adaptations after bariatric surgery, especially when occurring over a short period, leading to postprandial overexcretion of insulin. The mechanisms of this oversecretion appear to be multifactorial [29], and as recently discovered also mediated by interleukin 1-β [30].

At baseline, systolic blood pressure tended to be lower in patients with hypoglycemia. This might be driven by increased vagal activity or reflect lower concentrations of stress hormones preventing hypoglycemia in these patients. However, as using a single blood pressure measurement for screening is not recommendable due to possible fluctuation, we did not follow up on this in final ROC analyses.

While both HbA1c and weight loss achieved decent AUCs in the ROC analysis, combining the two into a score an even higher AUC of 0.776 was achieved. The score was calculated by dividing absolute weight loss by HbA1c. One of the major strengths of this score is its easy and wide applicability, as these parameters are usually assessed postsurgery in bariatric patients and HbA1c can be measured in most labs worldwide. By assessing medical history, hypoglycemia-related symptoms, and the score together in postbariatric patients, MMTs may be performed more targeted in the future.

## Limitations

One possible limitation of this study is the use of a liquid mixed-meal test with fixed carbohydrate loads as opposed to body weight adapted loads. However, the current body weight was not predictive for the occurrence of symptomatic hypoglycemia and the median weight was similar in patients of both groups. These data do not suggest that baseline body weight had a relevant effect on the outcome of the MMT. However, the main limitation is the relatively small single center dataset, which limited the statistical analyses to be adequately performed, as well as the lack of a validation cohort. Therefore, our results and the proposed scoring system will require future studies to confirm their external validity, clinical applicability, and generalizability.

## Conclusion

HbA1c, absolute, and relative postoperative weight loss differ at baseline in postbariatric patients with versus without symptomatic hypoglycemia during a standardized mixed-meal test. A proposed score of postoperative weight loss divided by HbA1c could predict the occurrence of PBH in a mixed-meal test. Future studies will provide insights into the generalizability of these results and the practicability of a wide use of our score as a screening tool in postbariatric patients.

## Supplementary Information

ESM 1 (DOCX 27 kb)

## Abbreviations

AUC: Area under the curve
HbA1c: Glycosylated hemoglobin A1c
MMT: Mixed-meal test
ROC: Receiver operating characteristic
T2DM: Type 2 diabetes mellitus
