# Supplementary material for: Predictors of Postprandial Hypoglycemia After Gastric Bypass Surgery: a Retrospective Case-Control Study
Source: Obes Surg. 2021 Feb 23;31(6):2497–502. doi: 10.1007/s11695-021-05277-1 (PMC8113281; doi:10.1007/s11695-021-05277-1)
Supplement: Supplementary file 1 — (DOCX 27 kb) [file 11695_2021_5277_MOESM1_ESM.docx]

### Supplemental Table 1: Univariate Predictive Accuracy of Continuous Variables

| **Parameter** | **AUC** | **Odds Ratio** | **95% CI** |
| --- | --- | --- | --- |
| Age | 0.507 | 0.997 | [0.982; 1.012] |
| Years since surgery | 0.635 | 1.008 | [0.889; 1.143] |
| Weight pre surgery | 0.604 | 1.002 | [0.996; 1.008] |
| Weight current | 0.549 | 0.999 | [0.991; 1.007] |
| Systolic blood pressure | 0.721 | 1.000 | [0.993; 1.007] |
| Diastolic blood pressure | 0.587 | 0.999 | [0.989; 1.01] |
| Heart rate | 0.542 | 1.001 | [0.991; 1.012] |
| Absolute weight loss | **0.724** | 1.012 | [0.995; 1.03] |
| Relative weight loss | 0.724 | 1.012 | [0.991; 1.033] |
| BMI pre surgery | 0.650 | 1.006 | [0.99; 1.021] |
| BMI current | 0.577 | 0.996 | [0.973; 1.019] |
| Baseline glucose | 0.544 | 0.967 | [0.841; 1.112] |
| Baseline insulin | 0.588 | 1.046 | [0.964; 1.135] |
| Baseline c-peptide | 0.502 | 1.001 | [1; 1.002] |
| HbA1c | **0.760** | 0.981 | [0.861; 1.116] |
| Hemoglobin | 0.667 | 1.000 | [0.994; 1.005] |
| C-reactive protein | 0.547 | 1.096 | [0.916; 1.312] |
| Glomerular filtration rate | 0.597 | 1.002 | [0.995; 1.008] |
| HOMA-IR | 0.590 | 1.260 | [0.86; 1.847] |
| HOMA-beta | 0.658 | 1.003 | [0.998; 1.007] |
| HOMA2-IR | 0.555 | 1.004 | [0.998; 1.009] |
| HOMA2-beta | 0.526 | 1.399 | [0.843; 2.32] |

### Supplemental Table 2: Sensitivity and Specificity for Various Thresholds

| **Absolute weight loss (kg)** | | | **HbA1c (%)** | | | **Combined Score (kg/%)** | | |
| --- | --- | --- | --- | --- | --- | --- | --- | --- |
| **Threshold** | **Sensitivity** | **Specificity** | **Threshold** | **Sensitivity** | **Specificity** | **Threshold** | **Sensitivity** | **Specificity** |
| 16 | 1 | 0 | 4.3 | 1 | 1 | 2.857143 | 1 | 0 |
| 21 | 0.947368 | 0 | 4.5 | 0.947368 | 1 | 3.859649 | 0.947368 | 0 |
| 22 | 0.947368 | 0.0625 | 4.6 | 0.842105 | 1 | 4.230769 | 0.947368 | 0.0625 |
| 25 | 0.894737 | 0.125 | 4.7 | 0.736842 | 0.9375 | 4.40678 | 0.894737 | 0.0625 |
| 26 | 0.842105 | 0.375 | 4.9 | 0.631579 | 0.9375 | 5 | 0.894737 | 0.3125 |
| 27 | 0.842105 | 0.4375 | 5 | 0.473684 | 0.75 | 5.102041 | 0.789474 | 0.3125 |
| 29 | 0.789474 | 0.4375 | 5.1 | 0.421053 | 0.6875 | 5.471698 | 0.789474 | 0.4375 |
| 30 | 0.789474 | 0.5625 | 5.2 | 0.315789 | 0.6875 | 6.666667 | 0.789474 | 0.6875 |
| 34 | 0.684211 | 0.5625 | 5.3 | 0.157895 | 0.5625 | 6.842105 | 0.736842 | 0.6875 |
| 35 | 0.631579 | 0.625 | 5.4 | 0.105263 | 0.375 | 6.862745 | 0.736842 | 0.75 |
| 36 | 0.578947 | 0.75 | 5.6 | 0.052632 | 0.3125 | 6.923077 | 0.684211 | 0.75 |
| 38.2 | 0.578947 | 0.875 | 5.7 | 0 | 0.3125 | 7.207547 | 0.684211 | 0.9375 |
| 39 | 0.526316 | 0.875 | 5.8 | 0 | 0.125 | 9.622642 | 0.368421 | 0.9375 |
| 45.7 | 0.473684 | 0.9375 | 5.9 | 0 | 0.0625 | 10.40816 | 0.368421 | 1 |
| 51 | 0.315789 | 0.9375 |  |  |  | 16.74419 | 0.052632 | 1 |
| 56 | 0.263158 | 1 |  |  |  |  |  |  |
| 72 | 0.052632 | 1 |  |  |  |  |  |  |
